# The implementation of a translational study involving a primary care based behavioral program to improve blood pressure control: The HTN-IMPROVE study protocol (01295)

**DOI:** 10.1186/1748-5908-5-54

**Published:** 2010-07-16

**Authors:** Hayden B Bosworth, Daniel Almirall, Bryan J Weiner, Mathew Maciejewski, Miriam A Kaufman, Benjamin J Powers, Eugene Z Oddone, Shoou-Yih D Lee, Teresa M Damush, Valerie Smith, Maren K Olsen, Daren Anderson, Christianne L Roumie, Susan Rakley, Pamela S Del Monte, Michael E Bowen, Jeffrey D Kravetz, George L Jackson

**Affiliations:** 1 Center for Health Services Research in Primary Care, Durham VAMC, Durham NC. USA; 2 Department of Medicine, Division of General Internal Medicine, Duke University, Durham NC. USA; 3 Department of Psychiatry and Behavioral Sciences & Center for Aging and Human Development, Duke University, Durham NC. USA; 4 Department of Biostatistics and Bioinformatics, Duke University, Durham NC. USA; 5 Department of Health Policy and Management, Gillings School of Global Public Health, University of North Carolina at Chapel Hill, Chapel Hill, NC. USA; 6 VA Stroke QUERI Center, VA HSRD Center of Excellence on Implementing Evidence-Based Practice, Roudebush VAMC; IU Center for Aging, Regenstrief Institute, Indianapolis, IN. USA; 7 Department of Medicine, VA Connecticut Healthcare System, West Haven, CT; Yale University School of Medicine New Haven, CT. USA; 8 VA Tennessee Valley Geriatric Research Education Clinical Center (GRECC), HSR&D Targeted Research Enhancement Program for Patient Healthcare Behavior, and Clinical Research Center of Excellence (CRCoE), and the Department of Medicine, Vanderbilt University, Nashville, TN. USA; 9 VA Tennessee Valley Healthcare System, National Quality Scholars Fellowship Program, VA Tennessee Valley Geriatric Research Education Clinical Center (GRECC), VA Tennessee Valley Healthcare System, Health Services Research (HSR), and the Department of Medicine, Vanderbilt University, Nashville, TN. USA

## Abstract

**Background:**

Despite the impact of hypertension and widely accepted target values for blood pressure (BP), interventions to improve BP control have had limited success.

**Objectives:**

We describe the design of a 'translational' study that examines the implementation, impact, sustainability, and cost of an evidence-based nurse-delivered tailored behavioral self-management intervention to improve BP control as it moves from a research context to healthcare delivery. The study addresses four specific aims: assess the implementation of an evidence-based behavioral self-management intervention to improve BP levels; evaluate the clinical impact of the intervention as it is implemented; assess organizational factors associated with the sustainability of the intervention; and assess the cost of implementing and sustaining the intervention.

**Methods:**

The project involves three geographically diverse VA intervention facilities and nine control sites. We first conduct an evaluation of barriers and facilitators for implementing the intervention at intervention sites. We examine the impact of the intervention by comparing 12-month pre/post changes in BP control between patients in intervention sites versus patients in the matched control sites. Next, we examine the sustainability of the intervention and organizational factors facilitating or hindering the sustained implementation. Finally, we examine the costs of intervention implementation. Key outcomes are acceptability and costs of the program, as well as changes in BP. Outcomes will be assessed using mixed methods (*e.g*., qualitative analyses--pattern matching; quantitative methods--linear mixed models).

**Discussion:**

The study results will provide information about the challenges and costs to implement and sustain the intervention, and what clinical impact can be expected.

## Background

Controlling hypertension improves cardiovascular and renal outcomes, and the mechanisms for achieving control including diet, exercise, and medications are well known and accepted. Despite the increased incidence of hypertension-related diseases, well-established evidence-based guidelines, and the availability of over 100 antihypertensive medications, approximately 25% to 40% of veterans with hypertension in 2007 did not have adequate blood pressure (BP) control (≥140/90 mmHg) [1].

To address this problem, the Department of Veterans Affairs (VA) healthcare system recently set a target of bringing 75% of hypertensive patients under effective BP control. To achieve this target, the VA needs to deploy evidence-based interventions that are effective, sustainable, and scalable for a large, complex healthcare delivery system. In prior research, our group has demonstrated the efficacy and cost-effectiveness of a nurse-delivered tailored behavioral self-management intervention in a population of hypertensive United States veterans [2]. Several VA facility leaders have expressed interest in using this intervention to reach the 75% target. Despite scientific evidence that the intervention works, these facility leaders and other potential adopters want to know: What will it take to implement the intervention successfully outside the context of a randomized controlled trial? When implemented 'in the real world,' will it produce the same results that it produced in the trial? What is necessary to sustain intervention delivery over time? Finally, what are the costs to implement and sustain the intervention?

In this article, we describe the design of a 'translational' study that implements an evidence-based nurse-delivered tailored behavioral self-management intervention to improve BP control as it moves from a research context to a dynamic practice context. Specifically, the study seeks to: identify organizational factors associated with effective implementation of the intervention in VA facilities; evaluate the clinical impact of the intervention when implemented outside the context of a randomized controlled trial; assess organizational factors associated with the sustained delivery of the intervention over time; and calculate cost of the intervention as it is implemented by VA facilities. Guided by innovation and organization theory, this mixed-methods study examines these issues in three sites implementing the behavioral self-management intervention and nine usual care sites. Study results will provide information about the challenges and costs of implementing and sustaining the intervention in primary care settings within large, complex healthcare delivery organizations and determine the clinical impact of the intervention.

## Methods

### Conceptual framework

**Figure 1:**
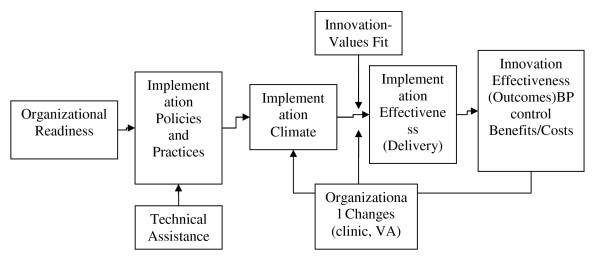
**Determinants of effective innovation implementation in organizations**.

To guide this evaluation project, we use an organizational model of innovation implementation (Figure 1) [3-6]. Briefly, the model posits that effective implementation of an intervention (*e.g*., consistent, high-quality, appropriate intervention delivery) is a function of organizational readiness for change; quality of the implementation policies and practices that the clinic puts into place; adaptations that the clinic makes to increase the fit of the intervention with clinic operations; the climate for implementation that results from these policies, practices, and adaptations; the extent to which intended users (*e.g*., physicians, nurses) perceive that the intervention reflects their values (*e.g*., professional autonomy, practice boundaries); and the extent to which clinic-level and organizational changes reinforce or reduce the climate for implementation (*e.g*., users' perceptions that intervention use is rewarded, supported, and expected). Effectiveness of the intervention (*e.g*., benefits, costs, and outcomes) depends, in part, on effective implementation. Effectiveness of the intervention, in turn, shapes users' perceptions that the intervention is worthwhile (rewarded, supported, and expected), which then affects the sustainability of the intervention.

### Overview of the intervention and its efficacy

The intervention is a nurse-delivered tailored telephone intervention that was developed and previously evaluated in the Veteran-Study To Improve The Control of Hypertension (V-STITCH) [7,8], and refined in Take Control of Your Blood (TCYB) Pressure study [9-11] and Hypertension Intervention Nurse Telemedicine Study (HINTS) [12]. In total, over the past eight years more than 1,800 hypertensive patients have been enrolled and followed for 18 to 24 months in a version of the behavioral-educational self-management intervention. The intervention is tailored to each patient's needs [13].

The intervention uses a behavioral-educational approach to enhance hypertensive patients' self-management capability and is organized around telephone encounters that occur approximately once every 4 to 5 weeks for 12 months. During the phone calls, trained nurses use the intervention software to gather medical and behavioral information. Patient responses to these questions activate a set of behavioral and educational modules within the intervention software that address such issues as social support, knowledge, health behaviors including smoking, weight loss, diet, alcohol use, stress, and participatory decision making [8,10,12].

### Overview of the implementation scheme

#### Setting of study

We have included three intervention sites located in three separate Veterans Integrated Service Networks (VISNs). Intervention facilities were selected based on four criteria. First, these facilities perceived that they could further benefit from improving the level of BP control at their facilities. Second, their patient demographics (rural versus urban, proportion of minorities) vary, which increases the generalizability of evaluation results. Third, the investigators have established collaboration with the leaders of these VISNs. Finally, the intervention sites agreed to leverage resources and funds to support a nurse (or nurses) required to implement the intervention. Each intervention site is matched to three control sites (nine in total) based on the level of VA organizational complexity and VISN affiliation.

#### Implementation parameters

Organizations often find it necessary and desirable to adapt evidence-based interventions to facilitate implementation, encourage ownership, and enhance acceptability among target populations [14]. The challenge for intervention developers is to encourage implementing sites to adapt the intervention to meet local needs and circumstances, yet discourage adaptations that undermine the intervention's 'active ingredients'--that is, the core elements of the intervention that embody its theory and internal logic, and produce its main effects [15-17].

We sought to balance the competing demands of adaptation and fidelity by requiring intervention sites to use certain intervention features and implementation processes while allowing them the flexibility to tailor other aspects of the intervention and the implementation process to local conditions, and providing intervention sites with centralized implementation support (Table 1). This approach allows us to incorporate lessons learned about successfully implementing interventions in organizational settings like the VA, to create enough comparability across implementing sites to support statistical and qualitative analysis, and to discover from the variability across implementing sites what works and what does not.

**Table 1:** Required elements and permitted adaptations to intervention features and implementation processes

Required elements	Permitted adaptations
Site implementation team must include designated 'innovation champion' and IT specialist.	Innovation champion can be nurse, physician, or manager.
Site implementation team must involve physicians, nurses, and administrators.	Implementation team structure and process (*e.g*., member roles, meeting frequency, and activities) can vary.
Site must commit one-half FTE for intervention position (*i.e*., the 'nurse').	Nurse can be registered nurse or other adequately trained clinician (*e.g*., pharmacist).
	Nurse position can be filled by one person or multiple people (totaling one-half FTE).
Site must enroll a minimum of 500 patients in the first 12 months of the implementation study period.	Sites can enroll patients through referral by primary care physicians or through pre-populated list by nurse.
Sites must establish a clinical reminder system that includes an option to order the intervention for patients with out of control hypertension (>140/90 mmHg).	The reminder may either be based on the VA electronic medical record system or a paper reminder from the clinic intake nurse for a given patient visit.
Sites need to notify provider if patient enrolled in program.	Methods for providing feedback to providers may vary by site.
Site must participate in centralized support activities.	Methods for communicating with central site may vary by site.

#### Facility implementation teams

Intervention facilities are required to commit at least four staff members in this partnership to ensure open communication among site participants and increase the likelihood of effective implementation: nurse interventionist, site principal investigator (physician), representative of the nursing administration, and information technology (IT) support staff. Each site has to agree to fund at least one-half of a full-time equivalent (FTE) nurse position, filled by one or more individuals. The nurse(s) will need to implement the program for two years--one year of enrollment and one year of follow-up. The facilities are responsible for determining nursing resources available to deliver the intervention, so these individuals may include both primary care staff nurses and individuals with experience as case managers.

The facility also is required to identify a specific site principal investigator, who leads the implementation effort at the facility and acts as a conduit between the facility and the centralized implementation support team. In the case of the present study, this person is typically a physician. In addition, participation requires the support of the director of nursing, who has the authority to dedicate nursing time for the intervention. Lastly, the site has to designate an information technology staff to be a contact and troubleshooter for the roll-out and use of the intervention software.

#### Patient enrollment

Each intervention facility has the goal of enrolling 500 patients during the 12-month implementation period. Patients can be referred to the intervention in any of the following three ways, depending on the preferences of the primary care providers at each intervention site:

1. For VA patients with a diagnosis of hypertension and last BP reading of >140/90 mmHg, primary care providers receive a reminder that the patient has poorly controlled hypertension that includes an option to place an order for the behavioral-educational intervention.

2. An item has been added to the providers' primary care screen in the VA electronic medical record that will allow a patient's provider to order the intervention even if the hypertension reminder has not been triggered for the patient.

3. If few intervention orders are received, the nurse is able to access a pre-populated list of patients who meet the same criteria as the hypertension care reminder. Starting with the patient with the most recent outpatient BP record, the nurse would contact the patient's primary care provider regarding the intervention.

#### Feedback to providers

Facilities can use one of two approaches to scheduling patients. In some cases, facilities have developed a specific nurse telephone hypertension self-management clinic established for the purpose of delivering the intervention. Like other healthcare appointments, the clerk receives an order from a primary care provider to schedule a specific time for the nurse to call the patient. The other option allows facilities to develop an alert that goes to the nurse indicating that a new patient is in the queue to be called. Upon calling the patient, the ordering provider is notified.

The nurse must place a note in the VA electronic medical record, the Computerized Patient Record System (CPRS), to describe any patient concerns. The nurse is responsible for addressing serious patient needs during the call following standard facility/clinic operating procedures.

#### Operating the intervention software

The intervention software is a distributed application built using the Microsoft .net framework. Users navigate to a VA intranet web page to launch the software. Using this system, nurses are able to access records from their site only. Data are transmitted within the VA protected computer environment (*i.e*., behind the VA firewall) using a point-to-point connection between the user's computer and a centralized server as the user goes through each screen that corresponds to call script and data collection.

#### Centralized support by intervention developers

Centralized support for the intervention is being provided to facilities by the research team. The support utilizes a number of processes from quality improvement collaboratives, such as those developed by the Institute for Healthcare Improvement (IHI) [18], including preliminary steps in which structured information is collected from facilities with the goal of helping them to plan for implementation. For example, facilities were sent worksheets asking them to identify team members, how the half FTE nurse would be acquired, and commitment signatures from the director of primary care and the director of primary care nursing. Monthly calls involving all team leaders have begun and will continue throughout the implementation period so that facilities can learn from one another's experience. Study staff has traveled to each facility to present information to physicians, mid-level providers, and other intervention staff as well as meet with facility leadership. Finally, the study project manager sends weekly reminders to facilities asking about meetings and workload for the economic analysis component of the study. This type of centralized support mirrors other quality improvement efforts of the VA [19,20].

#### Involvement of outside experts

Part of the implementation process consists of presentations of our intervention to an expert panel and our key stakeholders for review and comments. This implementation process (and its study) is being conducted with support of the VA Quality Enhancement Research Initiative (QUERI)) [21-23] program for stroke prevention and care. QUERI is the VA's program for bridging health services research and VA operations to study the processes for implementing innovations in the VA healthcare system. We also seek input from our advisory committee which consists of members representing leaders at both the local and VISN level and other key stakeholders including representatives from VA Central Office. In addition, this committee will help to disseminate the intervention, if it is shown effective, on a national level.

### Overview of the evaluation study design

**Figure 2:**
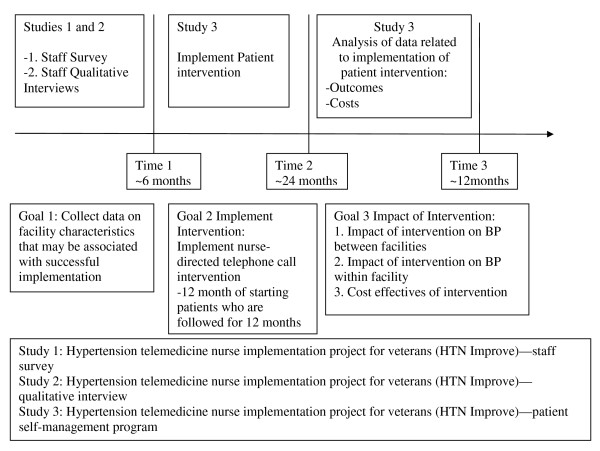
**Overall timeline**.

**Figure 3:**
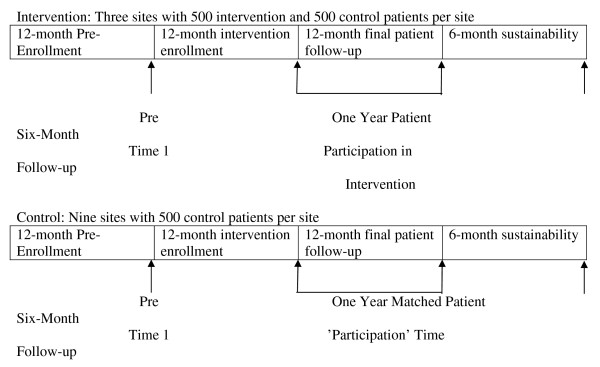
**Analytic study timeline for objective two**.

The remainder of this article describes four different components of the evaluation project that address implementation, clinical impact, sustainability, and costs of the behavioral-educational intervention. Table 2 summarizes major components of each study component. Figure 2 summarizes the overall study timeline. Figure 3 Summarizes the analytic study timeline for objective 2.

**Table 2:** Summary of study components

Aim	Research question	Unit analysis	Analysis methods	Outcome
1. Identify the organizational factors associated with the effective implementation of the intervention in VA facilities.	How do VA site leaders foster organizational readiness to implement the intervention? What VA clinic policies and practices are needed to support intervention use? Do VA clinics with a stronger implementation climate show more consistent, high-quality, appropriate intervention?	Organization (*e.g*., physicians, administrators, IT, nurses)	Qualitative/quantitative methods	An organizational model of implementation suitable for complex innovations and adapted to the context of clinical practice. While there are a number of methods available for implementing successful interventions, there lacks adequate examination of the most efficient methods for implementing this knowledge. An additional product of this phase of the study will be an evaluation of approaches to implementation of the behavioral intervention
2. Evaluate the clinical impact of the intervention when implemented outside the context of a randomized controlled trial.	What is the impact, in terms of average systolic BP improvement, of having implemented the behavioral intervention versus not having implemented the intervention as a facility-wide (*i.e*., clinical-level) program? Within sites that have implemented the behavioral intervention, what is the impact, in terms of average systolic BP improvement, of having received the intervention versus not having received the intervention?	Change in BP among those who receive the intervention relative to a comparison group of usual care	Quantitative methods	Demonstrate improved systolic BP in clinics using the intervention relative to clinics who did not receive the intervention
3. Assess the organizational factors associated with the sustained delivery of the intervention over time.	How do the perceived benefits and costs of the intervention affect the sustained use of the intervention by VA clinics? What policies and practices are necessary to support sustained use by clinics? How do organizational factors like staff turnover, competing priorities, and organizational changes affect sustained use by clinics?	VA clinics serve as the units of analysis. Focus on six VA clinics implementing the intervention. Data from the six VA clinics in the comparison group used to account for secular trends	Qualitative methods	Assess what implementation policies and practices are necessary to support sustainability and how organizational factors affect sustainability.
4. Calculate the cost of the intervention as implemented by VA facilities.	Do costs decline as the intervention moves from start-up and implementation to a steady state? Is the intervention cost-neutral or cost-saving? What is the value of implementing the intervention in VA clinics and the possible value of disseminating the intervention to other primary care settings.	Same sample used in study two to estimate costs	Quantitative methods	Detailed cost and resource estimates needed to implement the intervention will be available for all VA facilities.

### Study one objective: implementation study

Study one addresses the first specific aim: to assess organizational factors associated with the successful implementation of an evidence-based behavioral intervention to control BP. For this study, successful implementation of the intervention is defined by the degree to which patients receive scheduled phone calls that include presentation of content outlined by the intervention software. Informed by the conceptual model, study one research questions include: how do VA site leaders foster organizational readiness to implement the intervention; what VA clinic policies and practices are needed to support intervention use; and do VA clinics with a stronger implementation climate show more consistent, high-quality, appropriate intervention use as indicated by proxies such as patient retention, BP levels, and medication adherence? This component also seeks to describe the use of implementation approaches. While there are a number of methods available for implementing interventions, there is no consensus on the most efficient methods and dose of support for effectively implementing interventions [24].

### Design

Study one employs a case study design involving the collection and analysis of both qualitative and quantitative data. Case study methods are well-suited for studying implementation processes, which tend to be fluid, non-linear, and context-sensitive [25,26]. In addition to permitting in-depth analysis of individual cases, case study methods offer analytic strategies for systematically comparing patterns observed across cases [27]. The three VA clinics implementing the intervention serve as the units of analysis (*i.e*., the cases). Quantitative data from the nine VA clinics in the comparison group account for secular trends in hypertension management practices and clinical outcomes.

### Data collection strategy

Study one draws upon primary data collected from multiple sources using multiple methods to analyze potential facilitators and barriers to implementing the intervention, including site visits, semi-structured interviews, phone calls, e-mail exchanges, and standardized surveys. Prior to the launch of the intervention, we conduct interviews with the clinic director, physicians, nurses, IT staff, and office staff identified by the local site principal investigator who are involved in or affected by the implementation of the intervention (N = 42 to 60 total) (Table 3). We use a semi-structured interview guide to gather data on organizational readiness for change, implementation policies and practices, implementation climate, user-values fit, management support, and situational factors that might positively or negatively affect implementation success. Interviews will be audio-taped and transcribed verbatim. Monthly phone calls and discussion board exchanges with implementation group clinics will provide real-time data on what clinics are doing or not doing, what is working or not working, what clinics plan to do, and what assistance clinics need to support implementation. These data will not be audio-taped, but notes of the phone calls and discussion board exchanges will be summarized. These data provide supplemental information on management support, implementation policies and procedures, implementation climate, innovation-values fit, and other constructs.

**Table 3:** Anticipated sample size and composition for qualitative portion of the implementation survey

Role of Individual	N per VA site	Sample Total
Healthcare System		
Site Principal Investigator	1	3
Site Administrator	1	3
Site-Affiliated Physicians/healthcare providers	8 to 10	24 to 30
Site clinic staff members (*e.g*., secretaries, nurses, pharmacists)	3 to 5	9 to 15
Site Information Technology	1 to 3	3 to 9

**Total**	**14 to 20**	**42 to 60**

In addition to the wealth of qualitative data we plan to collect, we administer two surveys. The Assessment of Chronic Illness Care (ACIC) is implemented at baseline and 12 months at both the implementation clinics. The ACIC is developed to allow healthcare teams to evaluate the degree to which their organization has implemented practices suggested by the Chronic Care Model [28,29]. The ACIC has been shown to be responsive to quality improvement efforts [30,31]. We administer a web-based version of the survey to all primary care physicians, mid-level providers, and nurses, as well as selected administrators and IT specialists.

At the same time the ACIC is administered, the Organization Readiness to Change Survey is administered. Twelve items assess perceived efficacy of the core implementation group to carry out critical implementation tasks effectively (*e.g*., coordinating implementation activities), perceived commitment of the core implementation group to implement the intervention, and perceived commitment of the user group to support and use the intervention.

### Monitoring the intensity and dose of the behavioral intervention

Variation is expected in the procedures used by facilities to achieve the ability to deliver the phone calls as indicated by the intervention software. Thus, the following activities are used to fully capture the process for implementing the nurse-directed self-management support program:

1. We track the frequency of use of the intervention as well as number of written materials provided in the intervention sites.

2. We track the local variations and adaptations of our effective program at the intervention sites.

3. We track facility attendance on all study conference calls completed using a computer database to record the received dose of the patient intervention. This will provide us data on the consistency, quality, and appropriateness of intervention use.

### Analysis plan

Consistent with a case study research design, we use pattern-matching logic to guide data analysis [32]. In pattern-matching, an observed pattern is compared to a predicted one (*e.g*., hypothesized relationships shown in the conceptual model). If the patterns match, the predicted pattern is said to receive support. If the patterns do not, the investigator reformulates the predicted pattern by developing and investigating alternative predictions.

### Qualitative analysis

Procedurally, qualitative data analysis involves three phases: data coding, within-case analysis, and between-case analysis. In the first phase, we use qualitative data analysis software (ATLAS.ti 5.2) to code the study data. The conceptual model provides a starting list of codes, which we supplement with emergent codes as needed. In the second phase, we conduct a within-case analysis of each VA clinic implementing the intervention. Using ATLAS.ti, we generate reports of all text segments for each code. We assess the degree to which the construct appears in the data (its 'strength'), the degree to which the construct positively or negatively affects implementation (its 'valence'), and the degree to which relationships among constructs match the conceptual model.

### Quantitative analysis

Consistent with the organization-level focus of the conceptual model, we aggregate and analyze quantitative data at the VA clinic level (three intervention sites and nine control sites). We then analyze the quantitative data in conjunction with the qualitative data using the pattern-matching logic described above. For example, using the ACIC data, we examine whether VA clinics with more developed organizational infrastructures and climates supporting chronic care delivery at baseline exhibit greater management support, stronger implementation climates, better innovation-values fit, and more effective implementation. These data also help us gauge whether implementing the BP control intervention stimulated or facilitated more systemic changes in chronic care organization and delivery within the implementing clinic, or whether secular trends within the VA represent a plausible rival explanation for the results that we see.

### Products

Study one is expected to produce a theoretically informed, empirically grounded organizational model of implementation suitable for complex innovations and adapted to the context of clinical practice. An additional product of this phase of the study is an evaluation of approaches to implementation of the behavioral intervention.

### Study two objective: clinical impact

### Purpose

Study two seeks to assess the clinical impact of the implemented behavioral self-management intervention in order to assess the effectiveness of the intervention outside the supportive context of a randomized controlled trial. The population of interest is veterans with hypertension who meet criteria for the behavioral intervention and visit their primary care clinic at the VA for routine care. The two primary research questions are:

1. What is the impact, in terms of average systolic BP improvement, of having implemented the behavioral intervention versus not having implemented the intervention as a facility-wide (*i.e*., clinical-level) program?

2. Within sites that have implemented the behavioral intervention, what is the impact, in terms of average systolic BP improvement, of having received the intervention versus not having received the intervention (eligible but not approached for enrollment or eligible for enrollment but declined)?

Question one is an organizational (or policy) question that addresses the impact of rolling out the intervention facility-wide by comparing facilities implementing the behavioral intervention (implementation facilities) versus those that do not (control facilities). Question two addresses the impact of the intervention from the perspective of the patient by comparing patients receiving the intervention versus those that do not within facilities/clinics that implemented the intervention. Figure 2 summarized the time periods for which comparisons occur.

### Study design and sample

The study design is a clustered quasi-experimental (*i.e*., observational, non-equivalent groups) design with repeated measures [32]. Patients (the unit of analysis) are clustered within their facilities (clinics) and repeated BP measurements are gathered for each patient for over 12 months of participation in the intervention. The longitudinal design is unbalanced, meaning that BP values are not observed at distinct time points and not all patients contribute the same number of BP measurements. Due to logistics (*i.e*., hospital director approval, FTE requirements), for question one, clinics are not randomly assigned to implement versus not implement the behavioral intervention. Similarly, for question two, patients within facilities implementing the behavioral intervention are not randomly assigned to receive the intervention versus not receive the intervention.

For question one, the study sample includes all veterans with hypertension, who meet criteria for the behavioral self-management intervention, who visit participating clinics (both implementation and control clinics) at least three times in prior two years, and who have a BP measurement taken during the first visit. For question two, the study sample used to address question one is restricted to patients at implementation facilities.

### Measures

For both questions, the primary outcome is systolic BP, a continuous variable. Time is measured continuously in weeks since the first time a patient visits a participating facility during the implementation roll-out. For question one, the primary predictor variable is the implementation indicator variable (1 = implementation facility; 0 = control facility). For question two, the primary predictor variable is the treatment received indicator variable (1 = patient was contacted by nurse and received at least one phone call under the behavioral intervention; 0 = patient did not receive treatment).

### Data

This study relies primarily on data from the Veterans Health Information Systems and Technology Architecture (VistA), the electronic medical record system used to support both inpatient and outpatient care in the VA. Specifically, BP measurements (the primary outcome variable for both questions) and other covariates will be obtained from the Health Data Repository (HDR) for patients in our target population of interest. BP measurements in the HDR are date-stamped, allowing us to derive time (as defined above) for data analysis. The treatment received indicator variable (for question two) will be obtained from the software used by the study nurse to administer the behavioral self-management intervention.

### Confounding Bias

Because facilities are not randomized to implement or not implement the behavioral intervention (question one), and patients within implementation facilities are not randomized to receive or not receive the intervention (question two), an important challenge is the potential presence of confounding variables. A confounder variable is related to the outcome and is unevenly distributed between 'treatment' conditions (implementation for question one, and receiving treatment for question two), but is not in the causal pathway between the intervention and the outcome [33]. For question one, confounder variables may include facility-specific variables, such as the size of the facility, facility complexity, facility quality index, number of providers at the facility, a clinic's readiness to change, and other organizational factors measured prior to implementation roll-out. For question two, confounder variables may include patient-specific variables such as age, race, and clinical factors measured prior to receiving (or not receiving) the behavioral intervention--these include pre-intervention medication adherence, BP, hypertension concordant diagnoses (diabetes, kidney disease), or hypertension discordant diagnoses (chronic pain, mental illness). In order to minimize confounding bias for question two, possible confounder variables will be adjusted for in the data analyses.

### Data Analysis

For both questions, a linear mixed modeling (LMM) [34] strategy with random intercepts and slopes is used to estimate mean changes in BP over time, while taking into account the variability in BP for patients clustered within facilities [34]. With LMMs, patients are not required to have their repeated BP measurements taken over fixed time intervals throughout the study. All patients in the target population with at least one BP measurement are included in the data analysis. Therefore, the LMM is particularly suitable for this study given the unbalanced structure of the repeated BP measurements. This model is also known variously as a growth model or hierarchical linear model [35] for studying individual change within facilities; patients (level two units) with repeated BP measurements (level one) are nested within facilities (level three). Due to the relatively small number of implementation versus control facilities, the LMM will not accommodate the adjustment for all possible facility-level confounders of the impact of the implementation program on BP outcomes; therefore, confounding bias for question one will be examined qualitatively by interpreting the results of the LMM in light of how facilities differ on putative facility-level confounders. Putative patient-level confounders of the effect of treatment received on BP outcomes are included in the LMM for question two. For both questions, the primary outcome of interest is the mean difference in BP outcomes at 12 months, estimated using each of the LMMs.

### Statistical power and sample size considerations

Statistical power considerations are based on question one. Based on previous data [7,36], we anticipate that both implementation and control clinics have at least 500 hypertensive patients visit the clinic during the implementation roll-out period for which BP measurements are available (6,000 patients total). Due to the longitudinal nested study design (*i.e*., repeated systolic BP measurements on patients nested within clinics), clustering by clinic and within-person correlations must be taken into account in both the data analysis and power calculations. Following Donner and Klar [37], we use an inter-cluster correlation coefficient (ICC) and the correlation between repeated BP measurements to adjust the variance of a two-sample difference in means test (for the primary contrast of interest) in order to account for clustering and the longitudinal design, respectively. Most primary care clinical studies with a cluster design experience an ICC of approximately 0.01 to 0.05 [38]. Assuming an ICC equal to 0.01 and a correlation of 0.50 between baseline and 12-month systolic BP (these two assumptions are based on unpublished data from a previous study [2]), a two-tailed Type I error rate of 0.05, and given the sample size projections above, we expect to have 80% power to detect effect sizes that are at least as large as 0.22 (approximately a small effect size according to Cohen [39] for the mean difference in BP at 12 months). These calculations assume a balanced design (three implementation and three control sites) to simplify power calculations. Based on previous data suggesting a standard deviation of 18 mmHg in systolic BP [2,40], a minimum detectable effect size of 0.22 translates to a difference of approximately 4.0 mmHg in systolic BP between implementation sites and control clinics.

### Products

We anticipate one major product of the study to demonstrate improved systolic BP in clinics using the intervention relative to clinics who did not receive the intervention.

### Study three objective: sustainability study

Study three assesses the sustainability of the behavioral-educational self-management intervention to control BP. Just as it is necessary to study the processes through which patients must make a long-term commitment to self-management of hypertension, we study the ability of VA facilities to make long-term commitments to support the intervention. In this study, sustainability is operationalized as the willingness and capacity of VA facilities to maintain intervention use beyond the initial 12-month period in which new patients are enrolled and existing patients continue to receive the intervention. Specifically, three research questions are examined: How do the benefits and costs of the intervention as perceived by various stakeholders affect the sustained use of the intervention by VA clinics? What policies and practices are necessary to support sustained use by clinics? And how do organizational factors like staff turnover, competing priorities, and organizational changes affect sustained use by clinics?

### Design

Study three employs a case study design involving the collection and analysis of both qualitative and quantitative data. VA clinics serve as the units of analysis (*i.e*., the cases). The focus is on the three VA clinics implementing the intervention. Data from the nine VA clinics in the comparison group are used to account for secular trends in hypertension management practices and clinical outcomes.

### Data collection strategy

We obtain quantitative data from the intervention software on the clinics' actual use of the intervention beyond the initial 12-month enrollment period. As in study one, we obtain from the software data concerning the consistency, quality, and appropriateness of intervention use with respect to the 500 patients enrolled in the study. In addition, we examine whether clinics have enrolled new patients in the intervention and, if so, whether invention delivery is consistent, high-quality, appropriate in the expanded patient cohort.

### Analysis plan

Study three uses a pattern-matching logic in which the observed pattern of results is compared to the predicted pattern described in the conceptual model. Likewise, quantitative data are aggregated to the VA clinic level and analyzed in conjunction with qualitative data using pattern-matching logic. Using the ACIC data, we examine whether VA clinics with more developed organizational infrastructures and climates supporting chronic care delivery at baseline exhibit more sustained use of the BP intervention.

### Products

Results of study three allow us to assess what implementation policies and practices are necessary to support sustainability, and how organizational factors like staff turnover, competing clinic priorities, and larger organizational changes affect sustainability. Understanding the sustainability of the intervention is essential for ensuring the implementation of the program across the wider VA.

### Study four objective: healthcare costs

#### Purpose

Study four involves evaluating two types of costs usually assessed in randomized trials (costs associated with implementing the intervention and costs of veterans receiving the self-management intervention), and a third type of costs not assessed in randomized trials that relate to intervention dissemination to the clinics, which involves initial time costs of study investigators and clinic leadership in the buy-in and planning phases of the study [41]. Together, these three types of costs will create a complete picture of the costs that would be incurred if the intervention were adopted system-wide. Questions addressed with this aim include: Are per patient costs different between intervention sites and control sites? Do costs decline as the intervention moves from start-up to a steady state? Assessment of cost-analysis also provides useful information regarding the value of implementing the intervention in VA clinics and the possible value of disseminating the intervention to other primary care settings.

#### Study design and sample

The study sample for the cost analysis includes the matched cohorts of 6,000 veterans with hypertension who meet criteria for the behavioral self-management intervention and have at least one visit to the participating clinics during the intervention roll-out period and have a BP measurement taken during the first visit (total N = 6,000).

#### Intervention-related costs

This includes three costs: study investigator time modifying the intervention in preparation for the roll-out period; study investigator and clinic staff time spent during the buy-in phase developing trust and commitment to the implementation study; and study investigator and clinic staff time spent on implementing the intervention (*e.g*., planning, intervention training, nurse time) [41]. We are tracking the length of time for all meetings and other various communications to capture costs during the intervention modification and buy-in phases. To capture the amount of time the nurse spends on the phone with each patient, as well as the total amount of time spent documenting interactions with the patients, we utilize information on elapsed time that is captured automatically in the intervention software used by the nurses. Patient time spent on the telephone with the nurse also is obtained from the automatic elapsed time counter built into the software. To track nurse time spent communicating with providers and patients via email/fax/mail following the telephone intervention delivery, we provide the nurse with a spreadsheet to log these communications by patient and date. Costs for intervention supplies (computers, telephones) are based on their acquisition price from the manufacturer, and office space is calculated and allocated over their expected 'lifetime' of use.

#### Resource utilization and costs

Inpatient utilization data from the patient treatment file (PTF) data and outpatient utilization data from the Outpatient Care File (OPC) are to be merged with VA Decision Support System (DSS) data on VA expenditures for all trial participants to compare VA resource utilization of veterans randomized to treatment clinics and veterans randomized to control clinics before and during intervention roll-out. The outcome of interest is annual healthcare costs over the 12-month period, and the patient is the unit of analysis. A VA payor perspective is applied. All costs are to be valued in 2010 US dollars, based on the Current Price Index-Medical (CPI-M).

#### Products

We anticipate a major product of the study to outline the full range of costs required to implement and sustain the intervention in the six intervention clinics relative to clinics who did not receive the intervention.

#### Other issues

Conducting implementation research can be challenging and because of initial IRB challenges, we had to modify the study from six intervention sites and six control sites to the current three intervention sites compared to nine usual care sites.

## Discussion

The prevalence of hypertension has increased to 29.3% in 2003 to 2004 [42], resulting in 65 million Americans with hypertension (and upwards of 8.5 million veterans) and a greater burden of cardiovascular disease outcomes [42]. With increased prevalence of hypertension and subsequent secondary diseases, and poor control in treated patients, it is more important than ever to improve control of this prevalent disease.

Despite solid evidence of efficacy, there has long been a knowledge-practice gap in implementing hypertension interventions [43]. In addition, there has been inadequate attention regarding the sustainability of effective interventions. As such, studying the implementation of the behavioral-educational self-management intervention offers an opportunity to advance scientific knowledge about the challenges of intervention implementation and sustainability. Furthermore, an examination of organizational barriers and facilitators of evidence-based interventions may also help to improve the dissemination of evidence-based behavioral interventions for other chronic diseases.

The clinical strengths of our evaluation project include: building upon previously successful interventions that have resulted in improved BP control; an intervention that uses resources already available in primary care clinics and that could be redeployed in new ways to achieve higher quality of care for patients with hypertension; and assessing the costs associated with implementation of the intervention. Demonstrating the costs of the intervention will help ensure the dissemination of the intervention.

This implementation study capitalizes on the national healthcare system of the VA to systematically examine the local adoption of an effective program aimed to manage veterans' hypertension while informing implementation science. The goals of the intervention are aligned with the performance goals of the hospital administration as demonstrated with the leveraging of facility resources. Nonetheless, implementation of evidence based practices requires changes across the system, and this study is designed to facilitate and evaluate such changes.

The magnitude of the gap between discovery and delivery cannot be understated. Nor can we underestimate the gap between what we know and what we need to know in terms of promoting the use of evidence-based guidelines in primary care settings. Given the magnitude of the 'systems change' that may be required to meet hypertension guidelines, the project may also have a significant impact on veteran's health by helping the VA to accelerate the translation of scientific advances into large-scale improvements in health and substantial reductions in health disparities. Findings from the current endeavor may transcend the VA into other healthcare settings.

## Competing interests

The authors declare that they have no competing interests.

## Authors' contributions

HBB and GLJ were responsible for obtaining funding. All authors contributed to the design of the study, implementation of the project, design and coordination of the study, and helped to draft the manuscript. All authors read and approved the final manuscript.
